# Adaptation and validation of the chronic illness-related shame scale among patients with knee osteoarthritis in Singapore

**DOI:** 10.1186/s12891-023-06707-0

**Published:** 2023-08-04

**Authors:** Jia Ying Yeo, Chien Joo Lim, Bryan Yijia Tan

**Affiliations:** 1 Department of Rehabilitation Services, Yishun Health, National Healthcare Group, 90 Yishun Central, 768828 Singapore; 2 Department of Orthopedic Surgery, Woodlands Health, National Healthcare Group, 2 Yishun Central 2, 768024 Singapore; 3 Department of Orthopedic Surgery, Woodlands Health, National Healthcare Group, 11 Jalan Tan Tock Seng, 308433 Singapore

**Keywords:** Chronic illness shame, Validation, Singapore, Knee osteoarthritis

## Abstract

**Background:**

Osteoarthritis (OA) of the knee is one of the most common and disabling conditions worldwide. A neglected aspect of knee OA is its psychosocial impact, such as shame. However, assessment tools to measure shame among patients diagnosed with knee OA are lacking. In this study, the psychometric properties of the Chronic Illness-related Shame Scale **(**CISS) were evaluated among knee OA patients in Singapore.

**Methods:**

Adaptations were made to CISS for use among the knee OA population. An exploratory factor analysis (EFA) was performed to analyze the factor structure. Cronbach’s Alpha and corrected item-total correlations were used to evaluate the internal consistency. Spearman correlation coefficient was used to test the correlation between CISS and Patient Health Questionnaire-4 (PHQ-4) to determine the validity of the instrument.

**Results:**

The EFA yielded a one-factor structure, with an eigenvalue of 4.78 explaining 68.25% of variance. Cronbach Alpha was 0.92, which indicated good internal consistency. The Spearman correlation revealed a significant correlation between CISS and PHQ-4.

**Conclusions:**

The adapted CISS is a valid and reliable instrument to measure shame for knee OA patients. Both research and clinical settings can benefit from the use of the adapted CISS for assessing shame among knee OA patients.

## Background

Osteoarthritis (OA) is the most prevalent type of arthritis and one of the leading causes of disability among adults globally, with its prevalence increasing with age [1]. Of which, knee OA is the most common form, accounting for nearly 85% of the burden of osteoarthritis worldwide [2]. In Singapore, the national prevalence of knee OA is estimated at 11%, with a higher prevalence among women than men [3]. The prevalence of knee OA also increases with age, affecting 19.7% of those aged 60 years and above, compared to 8.6% of those between 18 and 59 years old [3].

Knee OA has a considerable impact on mobility, people’s quality of life, and daily living. However, the psychosocial impact of knee OA is often overlooked. On the social significance of OA, it has been highlighted that issues surrounding identity and biography play a key role to the meanings attached to illness and ageing [4]. Patients with chronic musculoskeletal pain condition were also found to have greater levels of shame compared to pain-free patients [5]. Being a chronic condition, knee OA may worsen over time, potentially becoming more visible to others [6]. It has been suggested that chronic disease patients, especially those with visible symptoms, are more likely to experience more shame [7]. A qualitative study in Singapore identified loss of face as a psychologic factor influencing the experiences of knee OA patients, describing slow gait speed and the use of walking aid as embarrassing and indicative of an illness [8], but few studies have measured the shame experienced by this group of people.

Shame is recognized to be a negative self-conscious emotion that is triggered by threats to an individual’s social identity and status [9]. The literature has suggested that any analysis of shame can be linked to two different types of interpretation – either externally focused or internally focused [10]. With external shame, the focus is on how individuals perceive themselves to exist negatively in the mind of others and believe that others view them as inferior, inadequate or flawed [11]. When these experiences are internalized, it can give rise to feelings of internal shame. For internal shame, the attention is more inwardly focused, and individuals may evaluate themselves negatively, deeming themselves as inferior, inadequate or flawed [11].

As researchers gain interest in measuring shame, various shame questionnaires have been developed, each with their own unique features. Some commonly used shame questionnaires include the Experiential Shame Scale (ESS) [12], the Test of Self-Conscious Affect (TOSCA-3) [13], and the Personal Feelings Questionnaire-2 (PFQ-2) [14]. However, these tools have mainly been studied among healthy college student population and have not specifically captured the experience of shame among chronic illness patients.

The lack of instruments measuring shame relating to chronic illness has prompted the development of the Chronic Illness-related Shame Scale (CISS), with constructs of internal and external shame as its theoretical framework [15]. Each item of the CISS was designed specifically to assess the level of shame experienced due to their chronic illness and/or its symptoms. While the scale has been validated, it was developed with a focus on patients with inflammatory bowel disease. Although the original study included a second sample of patients with other chronic illnesses, patients with knee OA were not reported to be part of the sample [15]. In addition, it was validated among Portuguese patients but has not been validated in the Asian context. Several factors are known to affect the magnitude and expression of a shame experience, some of which include the culture and background of the individual [16]. Thus, the purpose of this study is to explore the psychometric properties of the CISS adapted for the knee OA population in Singapore, by testing its factor structure, internal consistency, and validity.

## Methods

### Participants

This was a cross-sectional study with patients diagnosed with knee OA. A survey was conducted in two Singapore hospitals within the National Healthcare Group: the outpatient clinics of orthopedic and physiotherapy departments at Tan Tock Seng Hospital and Khoo Teck Puat Hospital. Patients were recruited using convenience sampling between June 2021 and February 2022.

This study involved a sample of patients diagnosed with knee OA who were aged 45 or older, independent community mobilizers with or without walking aids, conversant in English or Chinese, and met the clinical criteria for OA diagnosis as outlined by the National Institute for Health and Care Excellence (NICE). The NICE guidelines recommend that OA can be clinically diagnosed without investigations if an individual is aged 45 or above, has activity-related joint pain, and has either no morning joint-related stiffness or morning stiffness that lasts no longer than 30 min [17]. Exclusion criteria were any alternative diagnosis to knee OA (e.g., referred pain from the hip or spine), secondary arthritis (e.g., inflammatory, post-traumatic), inability to comply with the study protocol (e.g., cognitive impairment), previous knee arthroplasty, wheelchair user, pregnant women, and any other medical condition that would impair a participant’s ability to participate fully in the study (e.g., decompensated heart failure, stroke with significant residual functional weakness, psychiatric disorders such as psychosis, end-stage renal failure). Past medical records were reviewed, and the managing clinician was consulted whenever necessary to determine the eligibility of patients for the study.

### Data collection

Data for this study were drawn from a larger cohort study that explored the role of psychosocial factors on clinical outcomes and healthcare utilization among patients with knee OA [18]. Eligible patients were informed about the purpose of the study and consent was obtained. The questionnaires were either administered by the research coordinators at the patient’s preferred time and location or completed independently by the patient via an online digital secure form.

### Instruments

The CISS was used to measure chronic illness-related shame [15]. Items were adapted to reflect chronic knee pain condition experienced by the sample (e.g., I’m insecure due to my knee pain). The scale consists of 7 items rated on a 5-point Likert scale, ranging from 0 (Never True) to 4 (Always True). The final score was calculated by adding all the items. Higher scores indicate a higher level of shame associated with experiencing chronic knee pain and/or its symptoms. Psychometric properties of this instrument were also reported, demonstrating its reliability and validity [15]. The psychometric properties of the instrument were tested using two samples – one sample comprised of patients diagnosed with inflammatory bowel disease (further randomized into test group and validation group), and one sample comprised of patients diagnosed with at least one chronic illness. Results from the confirmatory factor analysis were good: root mean squared error of approximation (RMSEA) ranging from 0.06 to 0.10; comparative fit index (CFI) ranging from 0.96 to 0.99. The scale also exhibited excellent internal consistencies of 0.91 and 0.93, as well as composite reliability of 0.91 and 0.94.

The Patient Health Questionnaire-4 (PHQ-4) is an ultra-brief measure for screening both anxiety and depressive disorders [19]. There is vast empirical evidence suggesting that individuals who often experience feelings of shame about themselves were more prone to a variety of psychological symptoms, including anxiety and depression [20]. PHQ-4 [19] consists of a 2-item depression scale and a 2-item anxiety scale. The tool starts with the question of “Over the last 2 weeks, how often have you been bothered by the following problems?”, with responses scored as 0 (not at all), 1 (several days), 2 (more than half the days), or 3 (nearly every day). Total score was determined by adding the scores of all 4 items, resulting in a composite score ranging from 0 to 12. The scale has shown good internal consistency in previous studies (α > 0.80) [19, 21].

Demographic characteristics of the participants were collected using a structured questionnaire to gather information on gender, age, education level, employment status, number of years and the affected side of their knee OA.

### Sample size estimation

According to Tabachnick and Fidell [22], the required sample size for Exploratory Factor Analysis (EFA) is 150 subjects. Considering an anticipated attrition rate of 20%, the total sample size required was 150/ (1-0.2) = 188 respondents. As this was part of a larger cohort study, more participants than required were being recruited to ensure sufficient statistical power for other analyses and to account for a higher drop-out rate due to Coronavirus Disease 2019 (COVID-19).

### Statistical analyses

All data analyses were performed using IBM SPSS Statistics for Windows, Version 27.

#### Factor structure

To determine the suitability of the data for EFA, Kaiser-Meyer-Olkin (KMO) and Barlett’s test of sphericity were used. According to Kaiser and Rice [23], KMO values in the 0.90s are considered marvelous, in the 0.80s as meritorious, in the 0.70s as middling, in the 0.60s as mediocre, in the 0.50s as miserable, and anything less than 0.5 as unacceptable. The KMO cut-off value for this study was set at > 0.70. If Barlett’s test of sphericity is significant (significance level 0.05) and KMO value > 0.70, the data is deemed suitable for EFA.

The principal component method with varimax rotation was used to explore the factor structure of the CISS. Eigenvalues and scree plot were used to determine the number of components to be extracted. A widely used criterion is that of Kaiser [24] whereby only factors with eigenvalues greater than one were retained. The scree plot is a graphical method proposed by Cattell [25]. A scree plot generally has a sharp decline in magnitude of eigenvalues before levelling off, hence it is recommended to retain all eigenvalues in the sharp decline before the point where it starts to level off. Communalities and factor loadings were used to assess whether an item should be dropped from the scale. Items with low communalities would be dropped as they would likely not be related to any factor [26]. Any item with a communality score of less than 0.20 should be removed [27]. For factor loadings, scores greater than 0.40 were considered stable and should be kept [28].

#### Reliability of the CISS

Reliability analysis was carried out to assess the internal consistency of the CISS using Cronbach’s Alpha and corrected item-total correlations. Alpha values between 0.60 and 0.95 were considered acceptable and indicated good reliability [29]. The cut-off for corrected item-total correlations was set at 0.30 to indicate each item was related to the overall scale [30].

#### Convergent validity

To assess validity, the CISS sum score was used to measure the extent of association with PHQ-4. To choose the appropriate statistical analysis, normality of the CISS and PHQ-4 scores were checked using the histogram and boxplot. As significant deviation from normality was observed, the Spearman’s rank correlation was used.

### Ethical considerations

This study was approved by the Domain Specific Review Board of National Healthcare Group (WHC/2020-00076).

## Results

### Demographic characteristics

A total of 219 patients participated in this study. The age of participants ranged from 45 to 82 (M = 64.00 years, SD = 8.14). The mean duration (in years) of knee OA symptoms was 5.60 years (SD = 6.08). Most participants were female (68.9%), Chinese (82.2%), had up to secondary education level (44.3%), were employed (55.3%), and married (69.7%). Detailed demographic characteristics are presented in Table 1.

**Table 1:** Demographic information of participants

	No. of participants	%
Gender		
Male	68	31.1
Female	151	68.9
Age group		
45–59	68	31.1
60 and above	151	68.9
Race		
Chinese	180	82.2
Malay	15	6.8
Indian	21	9.6
Others	3	1.4
Highest education level		
No formal education	9	4.1
Elementary school	37	16.9
High school	97	44.3
Diploma holder	37	16.9
Degree holder	29	13.2
Others	10	4.6
Employment status^a^		
Employed	121	55.3
Unemployed	8	3.7
Homemaker	28	12.8
Retired	62	28.3
Marital status (n = 218)^b^		
Married	152	69.4
Divorced	17	7.8
Single	34	15.5
Widowed	15	6.8
Side of osteoarthritis (n = 218)^b^		
Left	46	21.0
Right	81	37.0
Both	91	41.6

^a^ Unemployed referred to participants who were not employed but actively seeking employment. Homemaker referred to participants who have been managing their household, were not employed outside the home, and not actively seeking employment; ^b^ Missing data resulted in n = 218 for marital status and side of osteoarthritis

213 responses were received for the CISS (97.3% of the participants), with scores ranging from 0 to 25 (M = 4.25, SD = 5.75). All participants answered the PHQ-4, with scores ranging from 0 to 12 (M = 1.53, SD = 2.44).

### Factor structure

The means and standard deviations for each item of the adapted CISS are presented in Table 2. The mean score for each item ranged from 0.38 to 0.84, implying that patients with knee OA tend to experience relatively low levels of shame about their condition. One-third of the sample (33.3%) scored above the mean total CISS score.

**Table 2:** Means and standard deviations for CISS

Item	Mean	SD
1. I feel isolated/alone due to my knee pain.	0.64	1.04
2. I’m ashamed of talking with others about my knee pain or symptoms.	0.38	0.78
3. I feel inferior and disregard myself because of my knee pain.	0.52	0.98
4. I feel that my knee pain is embarrassing.	0.46	0.91
5. I’m insecure due to my knee pain.	0.79	1.12
6. I feel that others may evaluate me negatively (or criticize me) due to my knee pain and symptoms.	0.61	1.03
7. I feel inadequate because of my knee pain and symptoms.	0.84	1.10

**Fig. 1:**
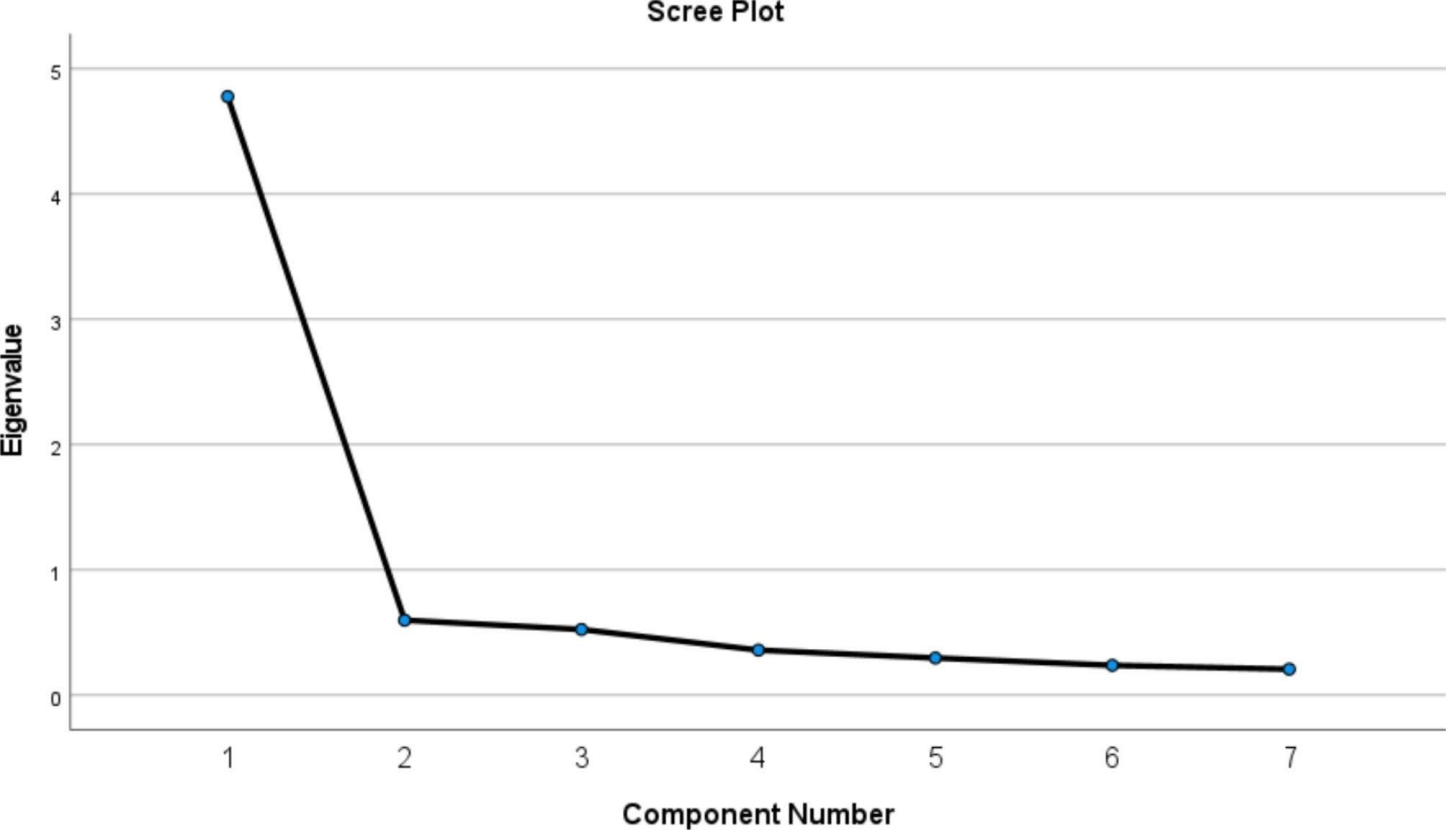
Scree plot of CISS. Only the eigenvalue for the first factor was greater than one and accounted for 68.25% of the total variance, indicating CISS had a unidimensional structure

The KMO measure of sampling adequacy indicated that the strength of the relationships among variables was high (KMO = 0.91), and Bartlett’s test of sphericity was significant (χ^2^[21] = 1012.43, *p* < 0.001). These results indicated that the data were suitable for EFA. The principal component method with varimax rotation with varimax rotation was performed and only one factor emerged, with eigenvalue of 4.78 which accounted for 68.25% of the variance observed. The scree plot is presented in Fig. 1. The communalities and factor loadings for each item of the adapted CISS are shown in Table 3.

**Table 3:** Communalities and factor loading of CISS

Item	Communalities	Factor loading	Cronbach alpha
1. I feel isolated/alone due to my knee pain.	0.61	0.78	0.92
2. I’m ashamed of talking with others about my knee pain or symptoms.	0.65	0.80	
3. I feel inferior and disregard myself because of my knee pain.	0.77	0.88	
4. I feel that my knee pain is embarrassing.	0.76	0.87	
5. I’m insecure due to my knee pain.	0.76	0.87	
6. I feel that others may evaluate me negatively (or criticize me) due to my knee pain and symptoms.	0.57	0.75	
7. I feel inadequate because of my knee pain and symptoms.	0.67	0.82	

The results revealed that the original one-factor structure was replicated among patients with knee OA in Singapore. One factor was extracted, and no items were removed from the model as all the items had communalities of more than 0.2 and factor loading of more than 0.4.

### Reliability

The internal consistency for the entire scale was excellent (α = 0.92). The corrected item-total correlations ranged from 0.67 to 0.83.

### Validity of instrument

**Fig. 2:**
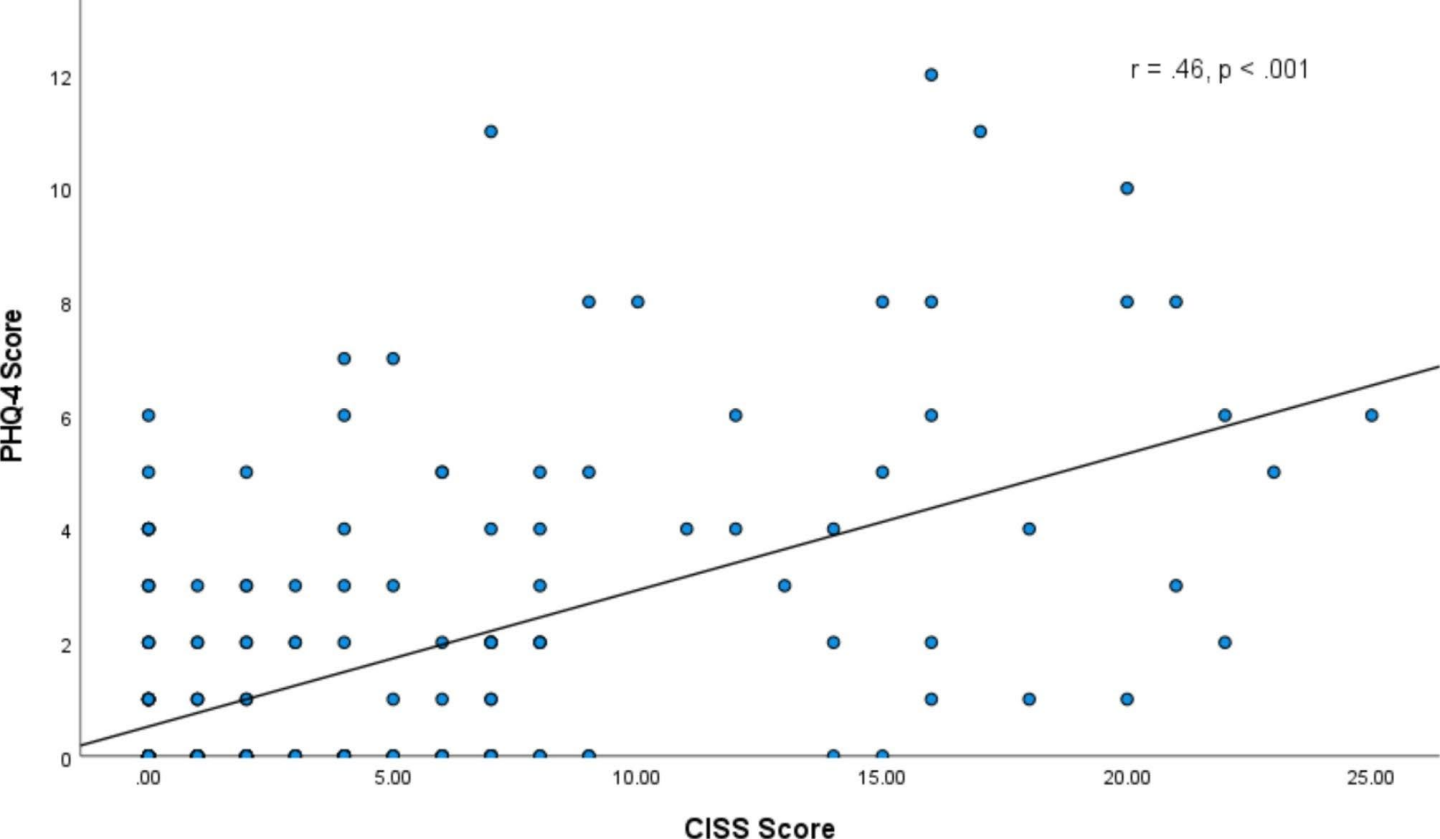
Scatterplot of CISS scores and PHQ-4 scores

The validity of the instrument was tested by examining the relationship between CISS and PHQ-4. A scatterplot depicting the relationship between CISS and PHQ-4 is shown in Fig. 2. The spearman rank correlation showed a significant positive correlation between the total scores of CISS and PHQ-4 (r_s_(211) = 0.46, *p* < 0.001), indicating those who experienced higher levels of shame also reported more anxiety and depression symptoms.

## Discussion

This study adapted the CISS and evaluated its psychometric properties for use among patients with knee OA in Singapore. The CISS is a scale developed to measure the level of shame experienced by patients due to their chronic illness and/or its symptoms. In this study, the EFA identified a unidimensional structure, which was consistent with the original study [15]. The scale showed good internal consistency from the Cronbach’s alpha score of 0.92 that was also similar to the original study [15]. Construct validity was demonstrated from its association with the PHQ-4 measuring depression and anxiety in the expected direction.

The role of cultural setting in shaping experiences of shame may be a factor to consider in interpreting the results of this study. According to Hofstede [31], both Portugal and Singapore are characterized as collectivistic societies, with Portugal having an individualism index of 27 and Singapore having an individualism index of 20. Wong and Tsai [32] made a comparison of the differences in triggers of shame between individualistic and collectivistic societies. They found that shame is elicited among individuals in individualist society in response to something that they did, whereas it is elicited among individuals in collectivistic society in response to actions of someone close to the individual. The inevitability of OA has been suggested to be a result of earlier life activities due to the overuse of their joints from working over the years or overactivity in sports [33]. This suggests that participants in this study may attribute their knee OA to their own past actions rather than the actions of others, thus possibly explaining the low sum scores observed in this study.

The feeling of shame is an overlooked and under-addressed emotion among knee OA patients. However, there is a significant impact of shame on their experience of pain and its management. The feelings of shame or embarrassment and being judged by others were felt by knee OA patients with the use of compensatory strategies that were visible, such as gait changes and using a walking aid [8]. The desire to appear independent and to not seem as having difficulty walking meant that they would choose not to use a walking aid as using it was a form of embarrassment [6]. To avoid judgement and hide their disability, they may also choose to withdraw from social interactions [8]. As feelings of shame could affect multiple aspects of the patient’s life, it is important for shame to be measured and understood in the clinical setting to allow clinicians to detect early whether shame may have a role in deciding subsequent management plans. Clinicians can then tailor treatment plans to suit the needs and lifestyle of the patient, such as involving a social worker for the psychosocial factors to be addressed or providing education on their condition. Thus, having a validated tool such as the CISS for the knee OA population will be beneficial for clinical purposes.

To our best knowledge, this is the first study that examined the psychometric properties of the scale for a sample of knee OA patients, as well as in the Asian context. Other scales such as the ESS and TOSCA-3 have been widely used. An advantage of the ESS is that it does not explicitly name shame, thereby reducing the tendency for subjects to put up defensive biases in reporting the feelings of shame [34]. On the other hand, instruments such as TOSCA-3 is a scenario-based measure that reduces the need to deny shame and places less reliance on the subject’s verbal skills, but it has been criticized for the lower internal consistency and the limited range of shame-inducing situations [34]. Despite the advantages of these instruments, they were not developed to target patients with chronic illnesses. It will thus be more appropriate to use CISS to measure shame for patients with chronic conditions such as knee OA. However, previous studies that used CISS were conducted in the Western countries such as Portugal [15] and Canada [35], all of which were sampled with inflammatory bowel disease patients. Little adaptation was made except for replacing the word illness with knee pain to better reflect the condition of the knee OA population. The good psychometric results from this study suggest that the scale can be adapted to a different culture without losing its validity for measuring shame. Thus, the good psychometrics properties of the adapted CISS demonstrated in this study can become a useful tool for the knee OA population.

Another strength of this study is the relatively large sample size. Recruiting patients from two different hospitals also allowed for a better representation of the knee OA population. Although the gender distribution in this study was predominantly females, this was expected as the prevalence of knee OA is higher among women than men [36].

These results must be interpreted in light of several limitations. Firstly, the population of patients with knee OA in this study reported relatively low levels of shame, which was observed from the mean score of all items ranging between 0.38 and 0.84. This is slightly lower compared to the original study where the mean score ranged from 0.90 to 1.71 [15]. Having a low baseline score reduces the sensitivity and responsiveness of the scale to capture changes in the level of shame experienced by patients with knee OA. Due to the busy nature of the clinic and the sampling method, this could have introduced selection bias. It is possible that we recruited a skewed sample of patients who were more well-adjusted and thereby more willing to participate in the study. This could have resulted in the low levels of shame experienced by the participants in this study. In addition, results may be different if those who declined participation were not as well-adjusted as those who agreed to participate. Secondly, confirmatory factor analysis (CFA) was not performed in this study. Even though there was a robust sample size for the EFA, we were not able to perform CFA as there were insufficient participants to form a second subsample. It will be beneficial for future study to perform CFA for further confirmation of the construct validity.

## Conclusion

This study has provided preliminary evidence on the psychometric properties of the adapted CISS, showing that the questionnaire is valid and reliable among patients with knee OA in Singapore. The adapted CISS has demonstrated good internal consistency and validity for assessing the level of shame experienced by knee OA patients. Factor analysis of the adapted scale revealed a one-factor structure, which was consistent with the original scale. Additional research is needed to explore the impact of shame on clinical outcomes among patients with knee OA and to determine whether any intervention should be warranted.

## Data Availability

The datasets used and analyzed during the current study are available from the corresponding author on reasonable request.

## Abbreviations

OA: Osteoarthritis
CISS: Chronic Illness-related Shame Scale
EFA: Exploratory Factor Analysis
PHQ-4: Patient Health Questionnaire-4
ESS: Experiential Shame Scale
TOSCA-3: Test of Self-Conscious Affect
PFQ-2: Personal Feelings Questionnaire-2
NICE: National Institute for Health and Care Excellence
RMSEA: Root Mean Square Error of Approximation
CFI: Comparative Fit Index
COVID-19: Coronavirus Disease 2019
KMO: Kaiser-Meyer-Olkin
SD: standard deviation
CFA: confirmatory factor analysis

## References

[1] Neogi T (2013). The epidemiology and impact of pain in osteoarthritis. Osteoarthr Cartil.

[2] Hunter DJ, Bierma-Zeinstra S, Osteoarthritis (2019). Lancet.

[3] Noviani M, Thumboo J, Lee CM, Soh I, Ma S, Leung YY (2016). SAT0463. The prevalence of knee osteoarthritis estimated by validated screening questionnaire in the general population of Singapore. Ann Rheum Dis.

[4] Sanders C, Donovan J, Dieppe P (2002). The significance and consequences of having painful and disabled joints in older age: co-existing accounts of normal and disrupted biographies. Sociol Health Illn.

[5] Turner-Cobb JM, Michalaki M, Osborn M (2015). Self-conscious emotions in patients suffering from chronic musculoskeletal pain: a brief report. Psychol Health.

[6] Trojanowski L. Experiences of stigma among people with osteoarthritis who underwent a total joint replacement of the knee or hip. Master’s thesis. Toronto: University of Toronto; 2019. Available from: https://hdl.handle.net/1807/96156.

[7] Matos-Pina I, Trindade IA, Ferreira C (2022). Internal and external shame in healthy and chronically ill samples: exploring links to psychological health. J Clin Psychol Med Settings.

[8] Yang SY, Woon EYS, Griva K, Tan BY (2023). A qualitative study of psychosocial factors in patients with knee osteoarthritis: insights learned from an asian population. Clin Orthop Relat Res.

[9] Gilbert P (2003). Evolution, social roles, and the differences in shame and guilt. Soc Res.

[10] Gilbert P, Tracy JL, Robins RW, Tangney JP (2007). The evolution of shame as a marker for relationship security: a biopsychosocial approach. The self-conscious emotions: theory and research.

[11] Gilbert P, Andrews B (1998). Shame: interpersonal behavior, psychopathology, and culture. Series in affective science.

[12] Turner JE. An investigation of shame reactions, motivation, and achievement in a difficult college course. Doctoral dissertation. Austin: The University of Texas at Austin; 1998.

[13] Tangney JP, Dearing RL, Wagner PE, Gramzow R. Test of self-conscious affect–3 (TOSCA-3). APA PsycTests. 2000. Accessed 10 Oct 2022.

[14] Harder DH, Zalma A (1990). Two promising shame and guilt scales: a construct validity comparison. J Pers Assess.

[15] Trindade IA, Ferreira C, Pinto-Gouveia J (2017). Chronic illness-related shame: development of a new scale and novel approach for IBD patients’ depressive symptomatology. Clin Psychol Psychother.

[16] Martens W (2005). A multicomponential model of shame. J Theory Soc Behav.

[17] National Institute for Health and Care Excellence. Osteoarthritis in over 16s: diagnosis and management. 2022. https://www.nice.org.uk/guidance/ng226/chapter/Recommendations. Accessed 12 Nov 2022. 36745715

[18] Tan BY, Goh ZZS, Lim CJ, Pereira MJ, Yang SY, Tan KG et al. Singapore knee osteoarthritis cohort (SKETCH): protocol for a multi-centre prospective cohort study. BMC Musculoskelet Disord. 2023 2023/02/07; 24(1):104. 10.1186/s12891-023-06207-1. 10.1186/s12891-023-06207-1 PMC9903549 36750930

[19] Kroenke K, Spitzer RL, Williams JBW, Löwe B (2009). An ultra-brief screening scale for anxiety and depression: the PHQ–4. Psychosomatics.

[20] Tangney JP, Stuewig J, Mashek DJ, Tracy JL, Robins RW, Tangney JP (2007). What’s moral about the self-conscious emotions?. The self-conscious emotions: theory and research.

[21] Mendoza NB, Frondozo CE, Dizon JIWT, Buenconsejo JU (2022). The factor structure and measurement invariance of the PHQ-4 and the prevalence of depression and anxiety in a southeast asian context amid the COVID-19 pandemic. Curr Psychol.

[22] Tabachnick BG, Fidell LS (2001). Using multivariate statistics.

[23] Kaiser HF, Rice J (1974). Little jiffy, mark IV. Educ Psychol Meas.

[24] Kaiser HF (1960). The application of electronic computers to factor analysis. Educ Psychol Meas.

[25] Cattell RB (1966). The scree test for the number of factors. Multivar Behav Res.

[26] Pituch KA, Stevens JP (2015). Applied multivariate statistics for the social sciences: analyses with SAS and IBM’s SPSS, sixth edition.

[27] Child D (2006). The essentials of factor analysis.

[28] Guadagnoli E, Velicer WF (1988). Relation of sample size to the stability of component patterns. Psychol Bull.

[29] Ursachi G, Horodnic IA, Zait A (2015). How reliable are measurement scales? External factors with indirect influence on reliability estimators. Procedia Econ Financ.

[30] Nunnally JC, Bernstein IH (1994). Psychometric theory. McGraw-Hill series in psychology.

[31] Hofstede G (2001). Culture’s consequences: comparing values, behaviors, institutions, and organizations across nations.

[32] Wong Y, Tsai J, Tracy JL, Robins RW, Tangney JP (2007). Cultural models of shame and guilt. The self-conscious emotions: theory and research.

[33] Smith TO, Purdy R, Lister S, Salter C, Fleetcroft R, Conaghan P (2014). Living with osteoarthritis: a systematic review and meta-ethnography. Scand J Rheumatol.

[34] Tangney JP, Dearing RL, Salovey P (2002). Shame and guilt.

[35] Mihajlovic V, Tripp DA, Jacobson JA (2021). Modelling symptoms to suicide risk in individuals with inflammatory bowel disease. J Health Psychol.

[36] Heidari B (2011). Knee osteoarthritis prevalence, risk factors, pathogenesis and features: part I. Casp J Intern Med.

